# Corneal nerve loss predicts dementia in patients with mild cognitive impairment

**DOI:** 10.1002/acn3.51747

**Published:** 2023-02-28

**Authors:** Georgios Ponirakis, Hanadi Al Hamad, Dunya A. M. Omar, Ioannis N. Petropoulos, Adnan Khan, Hoda Gad, Mani Chandran, Masharig Gadelseed, Ahmed Elsotouhy, Marwan Ramadan, Priya V. Gawhale, Marwa Elorrabi, Rhia Tosino, Ziyad R. Mahfoud, Shafi Khan, Pravija Manikoth, Yasmin H. M. Abdelrahim, Mahmoud A. Refaee, Noushad Thodi, Ahmed Own, Ashfaq Shuaib, Rayaz A. Malik

**Affiliations:** ^1^ Department of Medicine Weill Cornell Medicine‐Qatar, Qatar Foundation Doha Qatar; ^2^ Geriatric & Memory Clinic Rumailah Hospital, Hamad Medical Corporation Doha Qatar; ^3^ Neuroradiology Hamad General Hospital, Hamad Medical Corporation Doha Qatar; ^4^ MRI Unit Rumailah Hospital, Hamad Medical Corporation Doha Qatar; ^5^ Department of Medicine University of Alberta Edmonton Alberta Canada; ^6^ Faculty of Biology, Medicine and Health University of Manchester Manchester UK; ^7^ Faculty of Science and Engineering Manchester Metropolitan University Manchester UK

## Abstract

**Objectives:**

This study compared the utility of corneal nerve measures with brain volumetry for predicting progression to dementia in individuals with mild cognitive impairment (MCI).

**Methods:**

Participants with no cognitive impairment (NCI) and MCI underwent assessment of cognitive function, brain volumetry of thirteen brain structures, including the hippocampus and corneal confocal microscopy (CCM). Participants with MCI were followed up in the clinic to identify progression to dementia.

**Results:**

Of 107 participants with MCI aged 68.4 ± 7.7 years, 33 (30.8%) progressed to dementia over 2.6‐years of follow‐up. Compared to participants with NCI (*n* = 12), participants who remained with MCI (*n* = 74) or progressed to dementia had lower corneal nerve measures (*p* < 0.0001). Progressors had lower corneal nerve measures, hippocampal, and whole brain volume (all *p* < 0.0001). However, CCM had a higher prognostic accuracy (72%–75% vs 68%–69%) for identifying individuals who progressed to dementia compared to hippocampus and whole brain volume. The adjusted odds ratio for progression to dementia was 6.1 (95% CI: 1.6–23.8) and 4.1 (95% CI: 1.2–14.2) higher with abnormal CCM measures, but was not significant for abnormal brain volume.

**Interpretation:**

Abnormal CCM measures have a higher prognostic accuracy than brain volumetry for predicting progression from MCI to dementia. Further work is required to validate the predictive ability of CCM compared to other established biomarkers of dementia.

## Introduction

Dementia is a progressive neurodegenerative disease which currently affects 40–50 million people worldwide.^1^, ^2^ Early identification of individuals with mild cognitive impairment (MCI) at risk of progressing to dementia may enable earlier interventions. The 2018 revised NIA‐AA diagnostic criteria^3^ have proposed the use of cortical amyloid‐beta 42 (Aβ‐42) or tau PET, cerebrospinal fluid (CSF) Aβ‐42 or phosphorylated tau, and biomarkers of neurodegeneration, including CSF T‐tau, FDG PET hypometabolism, and atrophy on MRI to identify Alzheimer's dementia (AD) in its asymptomatic stages.^4^ The etiology of dementia is complex in terms of amyloid composition, tau distribution and their relationship to cognitive dysfunction. Furthermore, the prognostic ability of biomarkers for “pure” AD may be compromised by vascular pathologies,^5^, ^6^, ^7^, ^8^, ^9^ that may independently contribute to the development of dementia.^7^, ^10^, ^11^ Indeed, the Alzheimer's Drug Discover Foundation (ADDF) has emphasized the need for non‐amyloid biomarkers that are simple, non‐invasive, accurate and reliable for identifying individuals with MCI, at risk of dementia.

We have pioneered the technique of corneal confocal microscopy (CCM), a rapid non‐invasive ophthalmic imaging technique that enables quantification of corneal nerve morphology as a surrogate imaging marker for neurodegeneration in a range of peripheral neuropathies^12^ and central neurodegenerative diseases.^13^ We have previously shown evidence of corneal nerve loss in participants with MCI and dementia and demonstrated that CCM had a better diagnostic capability for identifying people with MCI compared to MRI brain volumetry.^14^, ^15^, ^16^ Furthermore, corneal nerve fiber loss was associated with the severity of cognitive dysfunction and functional independence^16^ as well as cerebral ischemia in patients with MCI and dementia.^7^

This study compared the ability of CCM with brain volumetry in identifying participants with MCI who progress to dementia.

## Materials and Methods

In this longitudinal cohort study, individuals with no cognitive impairment (NCI) and MCI aged 60–85 years were recruited from the geriatric and memory clinic in Rumailah Hospital, Qatar from 2016–2020 with a 1–4 year follow‐up from 2017–2021. CCM and brain MRI assessments were performed at baseline only and the follow‐up was undertaken in a routine clinic visit.

Exclusion criteria applied by geriatricians, geriatric psychiatrists and neurologists were dementia, Parkinson's disease, severe anxiety, severe depression, mood disorders, psychosis, hypomania, and peripheral neuropathy due to vitamin B_12_ deficiency and hypothyroidism. Self‐reported exclusion criteria included severe dry eye, ocular trauma or surgery in the preceding 12 months and corneal dystrophies were excluded after ophthalmic examination by the research investigators. Participants unable to cooperate during the CCM assessments were also excluded. Diabetes was not excluded as it is highly prevalent in patients attending the clinic.

This study was approved by the Institutional Review Board (IRB) of Weill Cornell Medicine in Qatar (WCM‐Q) and Hamad Medical Corporation (HMC) (IRB#: NPRP12S‐0213‐190080). All participants gave informed consent to take part in the study. The research adhered to the tenets of the declaration of Helsinki.

### Demographic and metabolic measures

Age, gender, blood pressure, body weight, body mass index (BMI), HbA1c, lipid profile, vitamin B_12_, thyroid function, and medical history were recorded from the electronic medical register (Cerner).

### Cognitive function assessment

Cognitive function was assessed using the Montreal Cognitive Assessment (MoCA) basic test version 7.1. A score of ≤26/30 indicates cognitive impairment.^17^ An extra point was added for individuals who were illiterate or had only attended primary school.

### Symptom severity rating

The severity of dementia symptoms was rated from 0 (normal), 0.5–1 (mild dementia), 2 (moderate dementia) and 3 (severe dementia) using the Clinical Dementia Rating (CDR) scale.^18^, ^19^ The ratings were based on direct interview and clinical judgment using a semi‐structured interview with the patient and a family member.

### Diagnosis

The diagnosis of MCI was based on ICD‐10: F06.7 criteria version: 2019. A consensus diagnosis was reached by geriatricians, geriatric psychiatrists and neurologists based on a comprehensive history and assessment of cognitive impairment using MoCA, psychiatric history, family and medical history, other medical comorbidities, medication and functional history of basic activities of daily living, dementia screening labs, alongside an MRI brain to exclude other causes of cognitive decline such as brain tumors, subdural hematoma, or normal pressure hydrocephalus.

The diagnosis of dementia, including AD, vascular dementia (VaD) and mixed AD was based on ICD‐10 criteria. The diagnosis of AD was based on typical symptoms and radiological features of AD e.g., atrophy in hippocampi, entorhinal cortex, and amygdala on MRI. Brain atrophy was assessed by neuroradiologists using the criteria of Dubois et al.^20^ blinded to the diagnosis and clinical data. The diagnosis of probable or possible VaD was based on the NINDS‐AIREN criteria,^21^ which include multiple large vessel infarcts or a single strategically placed infarct in the angular gyrus, thalamus, basal forebrain, or posterior or anterior cerebral artery territories, and multiple lacunes in the basal ganglia and white matter, extensive periventricular white matter lesions, or combinations thereof. The diagnosis of mixed dementia was based on the presence of AD and significant vascular changes.

### MRI brain volume analysis

MRI was performed on a 3T MRI system (MAGNETOM Skyra, Siemens AG, Erlangen). A T1‐weighted 3D MPRAGE sequence was obtained in the sagittal plane with a 1 mm slice thickness, repetition time of 1900 ms, echo time of 2.67 ms and 2.46 ms, inversion time of 1100 ms and 900 ms, flip angle of 9 degree and 15 degree, and FOV = 240 × 100. MRI brain volumetry was undertaken using NeuroQuant (NQ), FDA approved fully automated software.^22^, ^23^ The brain volume was adjusted for the percentage of intracranial volume (ICV) to minimize the impact of head size as a confounding factor. Thirteen brain structures, including the volume and ICV percentage of the hippocampus and whole brain were quantified.

### Corneal confocal microscopy

CCM analysis was performed in both eyes using the Heidelberg Retinal Tomograph 3 (HRT‐3) device with the Rostock Cornea Module (RCM) (Heidelberg Engineering GmbH, Heidelberg, Germany). The cornea was locally anesthetized by instilling 1‐drop of 0.4% benoxinate hydrochloride (Chauvin Pharmaceuticals, Chefaro, UK). Viscotears (Carbomer 980, 0.2%, Novartis, UK) was used as the coupling agent between the cornea and TomoCap, and between the TomoCap and objective lens. Patients were instructed to fixate on a target with the eye not being examined. High resolution 400 × 400 μm field of view images are generated using a 670‐nm red wavelength diode laser. Several scans of the sub‐basal nerve plexus in the central cornea were captured. To avoid bias, at a separate time, three high clarity non‐overlapping images per eye were selected based on depth, focus position and contrast as described previously^24^, ^25^, ^26^ by an investigator who was blinded from the diagnosis, cognitive function, and MRI brain volumetry. The central cornea was selected for imaging the sub‐basal nerve plexus. Corneal nerve fiber density (CNFD)‐ main fibers (no./mm^2^), corneal nerve branch density (CNBD)‐ branches from the main nerve fibers (no./mm^2^), corneal nerve fiber length (CNFL)‐ length of the main fibers and branches (mm/mm^2^), and CNBD/CNFD ratio were measured manually using CCMetrics.^27^

### Sample size calculation

We estimated that a minimum of 100 patients with MCI were required to estimate the area under the ROC curve (AUC) to determine the prognostic ability of corneal nerve measures or brain volumetry for progression from MCI to dementia over a mean follow‐up of 2‐years with a margin of error of at most 11% using 95% confidence intervals. This estimate incorporated the assumption of 30% progression to dementia^28^ and that the AUC will not be lower than 75%.

### Statistical analysis

Patient demographics and clinical characteristics were summarized using the mean and standard deviation for continuous variables and frequency distributions for categorical variables. Continuous and categorical variables were compared between patients with NCI and MCI who did and did not progress to dementia, using one‐way ANOVA and Chi‐square tests, respectively.

Receiver operating characteristic (ROC) curve analysis was used to determine the ability of corneal nerve measures and brain volumetry to distinguish between patients who did and did not progress to dementia. The area under the ROC curve (AUC) and the cut‐off value with optimal discriminative power for sensitivity and specificity, positive predictive value (PPV) and negative predictive value (NPV) were calculated using the Youden's index. The AUC of abnormal corneal nerve measures and brain volumetry versus abnormal corneal nerve measures and normal brain volumetry, normal corneal nerve measures and abnormal brain volumetry, and normal corneal nerve measure and normal brain volumetry was assessed.

In the binary logistic regression analysis, the dependent variables were progression from MCI to dementia, and the independent variables were abnormal corneal nerve measures or abnormal brain volumetry, age, sex, MoCA, education level, sleep deprivation, diabetes, duration of diabetes, obesity, hypertension, total cholesterol, triglyceride, HDL, systolic and diastolic blood pressure, weight, and BMI. Variables with *p* ≤ 0.05 at the bivariate level were included in the multiple logistic regression. Since listwise missing was used in this prior logistic regression, as a sensitivity analysis, the multiple logistic regression analysis was repeated where missing data for categorical MRI variables coded as unknown. Adjusted odds ratios (AOR), their corresponding 95% confidence intervals (CI) and *p* value are presented.

All analyses were performed using IBM‐SPSS (version 26; SPSS Inc, Armonk NY). A two‐tailed *p* value of ≤0.05 was considered significant.

## Results

### Clinical characteristics

Of 130 participants with MCI enrolled, 107 aged 68.4 ± 7.7 years underwent analysis, 3 dropped out due to incomplete assessments, and 20 were not included in the analysis as their follow‐up assessments were <12‐months from enrolment. Twelve age‐matched controls with no cognitive impairment (NCI) were recruited for baseline assessments only. All participants with MCI including those who developed dementia underwent follow‐up assessment in the clinic only, without CCM or MRI brain volumetry assessments. The follow‐up assessments were completed in 20 (19%) participants within 1–2 years, 35 (32%) participants within 2–3 years, 29 (27%) participants within 3–4 years, and 23 (22%) participants within 4–5 years of their baseline assessment.

Over a median follow‐up of 2.4 years, range 1–4.5 years, 33 (30.8%) progressed to dementia, and of the 74 (69.2%) who did not progress to dementia, 20 (18.7%) remained with MCI and 54 (50.5%) reverted to NCI.

Patients who progressed to dementia were significantly older (72.4 ± 6.3 vs 66.4 ± 8.0 years, *p* ≤ 0.0001), had a lower MoCA score (20.7 ± 6.2 vs 23.4 ± 4.7, *p* < 0.05), higher prevalence of hypertension (60.6% vs 38.4%, *p* < 0.05), shorter diabetes duration (7.3 ± 7.8 vs 12.3 ± 8.8 years, *p* < 0.05), lower triglyceride levels (1.3 ± 0.6 vs 1.7 ± 1.2 mmol/L, *p* = 0.01), and higher HDL levels (1.4 ± 0.5 vs 1.2 ± 0.4 mmol/L, *p* < 0.05) compared to those who did not progress. However, there were no differences in education level between those who completed primary school only (15.6% vs 15.5%), secondary school (78.1% vs 71.8%) or college (6.3% vs 12.7%) (*p* = 0.62), percentage of males (40.5% vs 45.5%, *p* = 0.63), sleep deprivation (61.9% vs 58.1%, *p* = 0.76), obesity (31.3% vs 42.4%, *p* = 0.29), weight (82.6 ± 16.7 kg vs 78.2 ± 22.9 kg, *p* = 0.33), BMI (31.1 ± 6.8 kg/m^2^ vs 29.4 ± 7.8 kg/m^2^, *p* = 0.30), diabetes (51.5% vs 51.4%, *p* = 0.99), systolic (137.1 ± 19.1 mmHg vs 141.0 ± 18.1 mmHg, *p* = 0.32) and diastolic blood pressure (72.0 ± 9.4 mmHg vs 72.2 ± 8.7 mmHg, *p* = 0.95), HbA1c (7.1 ± 1.7% vs 6.7 ± 1.6%, *p* = 0.31) and total cholesterol levels (4.5 ± 1.3 mmol/l vs 4.4 ± 0.8 mmol/l, *p* = 0.61) between participants with MCI who did and did not progress to dementia.

### Corneal confocal microscopy

**Figure 1:**
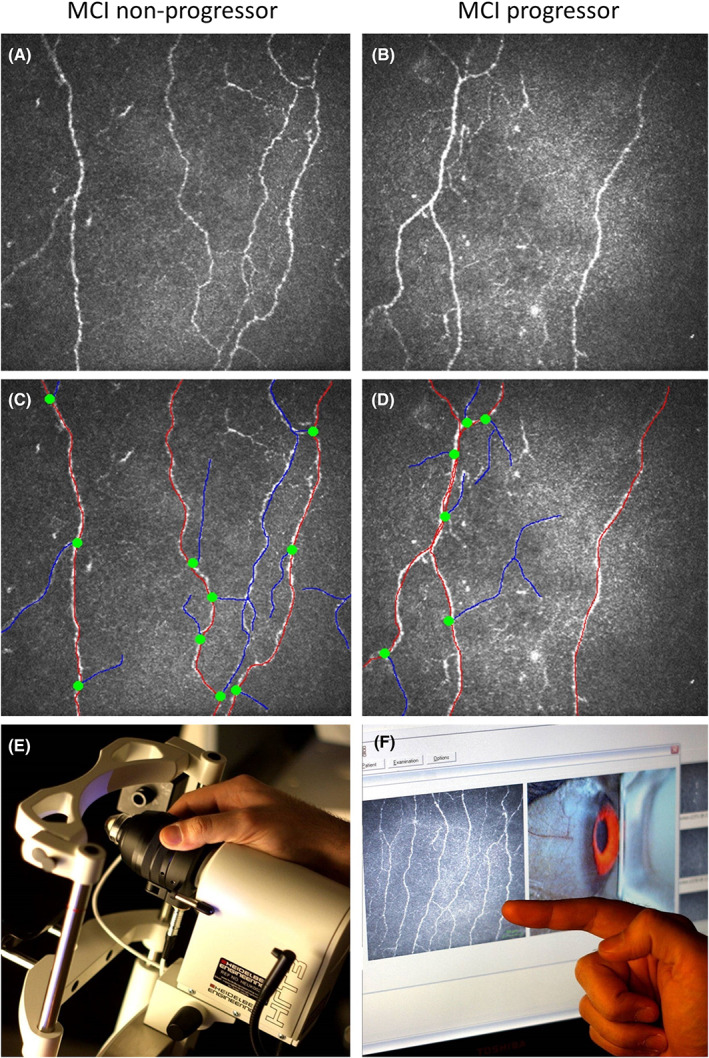
Corneal nerve fiber morphology in a patient with MCI with and without progression to dementia. Corneal confocal microscopy (CCM) images of the sub‐basal nerve plexus (A and B) and analyzed CCM images (C and D) from a patient with MCI without (A and C) and with (B and D) progression to dementia. The Heidelberg Retinal Tomograph 3 device with the Rostock Cornea Module (E) that captures the corneal nerve fiber images (F).

Participants with MCI who progressed to dementia (*n* = 33, 30.8%) had a significantly lower CNFD (20.6 ± 9.3 vs 28.8 ± 8.2 no./mm^2^, *p* < 0.0001), CNBD (43.3 ± 28.9 vs 70.8 ± 37.2 no./mm^2^, *p* < 0.0001), CNFL (14.3 ± 6.3 vs 19.9 ± 5.7 mm/mm^2^, *p* < 0.0001), and CNBD/CNFD ratio (1.9 ± 1.0 vs 2.4 ± 1.1, *p* < 0.05) at baseline, compared to those who did not progress (*n* = 74, 69.2%) (Table 1 and Fig. 1).

**Table 1:** Comparison of clinical characteristics, cognitive function, corneal confocal microscopy measures and MRI brain volumetry between patients with mild cognitive impairment who did and did not progress to dementia.

	Controls with NCI	MCI non‐progressors	MCI progressors	*p* value
*n* (%)	12 (N/A)	74 (69.2)	33 (30.8)	
*Clinical characteristics*				
Age, years	71.1 ± 6.0	66.4 ± 8.0*	72.4 ± 6.3	<0.0001
Male, *n* (%)	10 (83.3)	44 (59.5)	18 (54.5)	0.21
Diabetes, *n* (%)	N/A	38 (51.4)	17 (51.5)	0.98
Duration of diabetes, years	N/A	12.3 ± 8.8	7.3 ± 7.8	<0.05
Hypertension, *n* (%)	6 (50.0)	28 (38.4)	20 (60.6)	<0.05
Systolic blood pressure, mmHg	137.9 ± 18.7	137.1 ± 19.1	141.0 ± 18.1	0.32
Systolic blood pressure, mmHg	74.1 ± 12.6	72.0 ± 9.4	72.2 ± 8.7	0.96
BMI, kg/m^2^	27.2 ± 3.2	31.1 ± 6.8	29.4 ± 7.8	0.25
HbA1c, %	5.7 ± 0.4	7.1 ± 1.7**	6.7 ± 1.6	0.30
Total Cholesterol, mmol/L	5.3 ± 0.8	4.5 ± 1.3	4.4 ± 0.8	0.66
Triglycerides, mmol/L	1.6 ± 0.7	1.7 ± 1.2	1.3 ± 0.6	0.01
HDL, mmol/L	1.4 ± 0.4	1.2 ± 0.4	1.4 ± 0.5	<0.05
MoCA score	27.1 ± 5.2	23.4 ± 4.7*	20.7 ± 6.2****	<0.05
*Corneal confocal microscopy measures*				
CNFD, no./mm^2^	35.6 ± 5.8	28.8 ± 8.2**	20.6 ± 9.3****	<0.0001
CNBD, no./mm^2^	100.3 ± 47.6	70.8 ± 37.2**	43.3 ± 28.9****	<0.0001
CNFL, mm/mm^2^	25.3 ± 4.2	19.9 ± 5.7**	14.3 ± 6.3****	<0.0001
CNBD/CNFD ratio	2.9 ± 1.1	2.4 ± 1.1	1.9 ± 1.0**	<0.05
*MRI brain volumetry*				
Whole Brain, ICV%	73.5 ± 3.3	72.6 ± 3.1	69.0 ± 3.6***	<0.0001
Cortical gray matter, ICV%	29.6 ± 2.9	29.2 ± 3.6	27.3 ± 3.3	<0.05
Ventricle, ICV%	3.0 ± 1.5	2.9 ± 1.6	3.7 ± 1.5	<0.05
Hippocampus, ICV%	0.47 ± 0.05	0.45 ± 0.08	0.38 ± 0.06**	<0.0001
Entorhinal cortex, ICV%	0.38 ± 0.10	0.34 ± 0.11	0.31 ± 0.06	0.17
Thalamus, ICV%	0.94 ± 0.09	0.93 ± 0.14	0.87 ± 0.08	<0.05
Amygdala, ICV%	0.20 ± 0.02	0.19 ± 0.03	0.18 ± 0.03	<0.05
Brainstem, ICV%	1.48 ± 0.19	1.43 ± 0.18	1.41 ± 0.13	0.57
Cingulate gyrus, ICV%	0.99 ± 0.10	0.94 ± 0.20	0.95 ± 0.11	0.71
Frontal lobe, ICV%	10.6 ± 0.9	10.4 ± 2.0	9.8 ± 1.3	0.14
Temporal lobe, ICV%	7.7 ± 1.0	7.5 ± 1.4	7.1 ± 1.0	0.20
Parietal lobe, ICV%	6.7 ± 0.7	6.4 ± 1.3	6.1 ± 0.8	0.26
Occipital lobe, ICV%	3.7 ± 0.6	3.4 ± 0.9	3.4 ± 0.5	0.66

Variables presented as mean ± standard deviation were compared using one‐way ANOVA. Variables that were significantly different between controls and participants with MCI were denoted as *p ≤ 0.05, **p ≤ 0.01, ***p ≤ 0.001, ****p ≤ 0.0001.

CNBD, corneal nerve branch density; CNFD, corneal nerve fiber density; CNFL, corneal nerve fiber length; ICV, intracranial volume; MCI, mild cognitive impairment; MoCA, Montreal cognitive assessment; NCI, no cognitive impairment.

Compared to participants with NCI, both progressors and non‐progressors had significantly lower CNFD, CNBD, and CNFL (*p* ≤ 0.01–0.0001). The CNBD/CNFD ratio was significantly lower in those who progressed to dementia (*p* ≤ 0.01) but comparable in those who did not progress to dementia (*p* = 0.17) compared to those with NCI.

Participants with MCI who reverted to NCI (*n* = 54, 50.5%) had a higher CNFD (29.5 ± 8.3 vs 26.2 ± 7.9 no./mm^2^), CNBD (72.7 ± 40.3 vs 64.9 ± 27.9 no./mm^2^), and CNFL (20.3 ± 5.9 vs 18.5 ± 4.8 mm/mm^2^) compared to those who remained with MCI (*n* = 20, 18.7%); however, these differences were not statistically significant (*p* = 0.14–0.39). Compared to those with NCI, participants with MCI who reverted to NCI had significantly lower CNFD (*p* < 0.05), CNBD (*p* < 0.05) and CNFL (*p* < 0.01) with a comparable CNBD/CNFD ratio (*p* = 0.13).

### Brain volumetry

Participants with MCI who progressed to dementia had a significantly lower volume of whole brain (69.0 ± 3.6 vs 72.6 ± 3.1 ICV%, *p* < 0.0001), cortical gray matter (27.3 ± 3.3 vs 29.2 ± 3.6 ICV%, *p* < 0.05), hippocampi (0.38 ± 0.06 vs 0.45 ± 0.08 ICV%, *p* < 0.0001), thalamus (0.87 ± 0.08 vs 0.93 ± 0.14 ICV%, *p* < 0.05) and amygdala (0.18 ± 0.03 vs 0.19 ± 0.03 ICV%, *p* < 0.0001), and higher ventricle volume (3.7 ± 1.5 vs 2.9 ± 1.6 ICV%, *p* < 0.05) at baseline, compared to those who did not progress (Table 1). However, there was no difference in the volume of entorhinal cortex (*p* = 0.17), brain stem (*p* = 0.57), cingulate gyrus (*p* = 0.71), frontal lobe (*p* = 0.14), temporal lobe (*p* = 0.20), parietal lobe (*p* = 0.26), and occipital lobe (*p* = 0.66) between the two groups.

Compared to participants with NCI, the volume of whole brain (69.0 ± 3.6 vs 73.5 ± 3.3 ICV%, *p* < 0.0001), and hippocampi (0.38 ± 0.06 vs 0.45 ± 0.08 ICV%, *p* < 0.0001) were significantly lower in those who progressed to dementia, but comparable to those who did not progress to dementia (*p* = 0.41–0.52).

Participants with MCI who reverted to NCI had comparable volume of whole brain (72.6 ± 3.0 vs 72.6 ± 3.5 ICV%, *p* = 0.96) and hippocampi (0.46 ± 0.07 vs 0.44 ± 0.10 ICV%, *p* = 0.47) compared to those who remained with MCI. Compared to participants with NCI, those with MCI who reverted to NCI had a comparable volume of whole brain (*p* = 0.55) and hippocampi (*p* = 0.77).

### The predictive accuracy of CCM versus brain volumetry

The area under the curve (AUC) to predict progression to dementia was 74% (95% CI 64–85%, *p* < 0.0001) for CNFD, 73% (95% CI 63%–84%, *p* < 0.0001) for CNBD, 71% (95% CI 61%–82%, *p* < 0.0001) for CNFL, 63% (95% CI 51%–74%, *p* < 0.05) for the CNBD/CNFD ratio, 78% (95% CI 67%–89%, *p* < 0.0001) for hippocampal volume, 78% (95% CI 66%–91%, *p* < 0.0001) for whole brain volume (Table 2 and Fig. S1) and ranged from 63%–68% for ventricular, frontal lobe, amygdala, cortical gray matter, and thalamic volumes. Temporal, parietal, occipital lobe, entorhinal cortex, brainstem, and cingulate gyrus volumes were unable to predict progression to dementia.

**Table 2:** The predictive ability of corneal confocal microscopy and MRI brain volumetry measures for progression from MCI to dementia.

	AUC % (95% Cl)	*p* value	Cutoff point	Sensitivity (%)	Specificity (%)	Accuracy (%)	PPV (%)	NPV (%)
Hippocampus, ICV%	78 (67–89)	<0.0001	≤0.44	86	62	69	50	91
Whole brain, ICV%	78 (66–91)	<0.0001	≤72	86	60	68	49	90
CNFD, no./mm^2^	74 (64–85)	<0.0001	≤25	67	78	75	58	84
CNFL, mm/mm^2^	73 (63–84)	<0.0001	≤15	55	84	75	60	81
CNBD, no./mm^2^	71 (61–82)	<0.0001	≤44	55	80	72	55	80
Ventricle, ICV%	68 (55–82)	<0.01	≥3.0	71	68	69	50	84
Frontal lobe, ICV%	68 (55–80)	<0.01	≤10.8	86	52	62	44	89
Amygdala, ICV%	67 (53–80)	0.01	≤0.17	52	76	69	49	78
Cortical gray matter, ICV%	65 (52–78)	<0.05	≤29	71	54	59	41	81
Thalamus, ICV%	65 (52–78)	<0.05	≤0.90	86	54	64	45	89
CNBD/CNFD ratio	63 (51–74)	<0.05	≤2.54	82	42	54	39	84
*Unable to predict progression to dementia*								
Temporal lobe, ICV%	62 (48–76)	0.08	N/A	N/A	N/A	N/A	N/A	N/A
Parietal lobe, ICV%	61 (47–74)	0.12	N/A	N/A	N/A	N/A	N/A	N/A
Entorhinal cortex, ICV%	58 (45–71)	0.24	N/A	N/A	N/A	N/A	N/A	N/A
Occipital lobe, ICV%	56 (41–71)	0.08	N/A	N/A	N/A	N/A	N/A	N/A
Brainstem, ICV%	51 (36–65)	0.92	N/A	N/A	N/A	N/A	N/A	N/A
Cingulate gyrus, ICV%	49 (35–64)	0.92	N/A	N/A	N/A	N/A	N/A	N/A

Receiver operating characteristic (ROC) curve analysis.

CNBD, corneal nerve branch density; CNFD, corneal nerve fiber density; CNFL, corneal nerve fiber length; NPV, negative predictive value; PPV, positive predictive value.

The sensitivity, specificity, positive and negative predictive value were 67%, 78%, 58%, 84% for CNFD ≤25 no./mm^2^, 55%, 80%, 55%, 80% for CNBD ≤44 no./mm^2^, 55%, 84%, 60%, 81% for CNFL ≤15 mm/mm^2^, 86%, 62%, 50%, 91% for hippocampal volume ≤0.44 ICV%, and 86%, 60%, 49%, 90% for whole brain volume ≤ 72.0 ICV%, respectively. CCM measures had a higher prognostic accuracy for identifying participants who progressed to dementia compared to hippocampus and whole brain volume (72%–75% vs 68%–69%). There was a high percentage of abnormal CNFD (*n* = 20/55, 36.4%), CNBD (*n* = 19/55, 34.5%), and CNFL (*n* = 21/55, 38.2%) in those with a short follow‐up period of ≤2‐years (51.4%, *n* = 55/107).

The AUC of a combined abnormal CNFD (≤25 no./mm^2^) and abnormal hippocampus volume (≤0.44 ICV%) or abnormal whole brain volume (≤72 ICV%) were 75.6% (95% CI 63%–88%) and 72.4% (95% CI 60%–85%), respectively. The AUC of combined abnormal CNFL (≤15 mm/mm^2^) and abnormal hippocampus volume (≤0.44 ICV%) or abnormal whole brain volume (≤72 ICV%) were 71.9% (95% CI 59%–85%) and 69.5% (95% CI 56%–83%), respectively.

### Association of progression to dementia with abnormal CCM measures and brain volumetry

The odds ratio (OR) for progression from MCI to dementia adjusted for hypertension, triglycerides, HDL and diabetes duration was 6.3 times (95% CI: 2.0–20.2) higher with abnormal CNFD (≤25 no./mm^2^), 5.8 times (95% CI: 1.9–18.1) higher with abnormal CNBD (≤44 no./mm^2^), 4.4 times (95% CI: 1.5–13.2) higher with abnormal CNFL (≤15 mm/mm^2^), 6.4 times (95% CI: 1.4–30.0) higher with abnormal hippocampus volume (≤0.44 ICV%), and 7.5 times (95% CI: 1.4–40.9) higher with abnormal whole brain volume (≤72 ICV%).

**Figure 2:**
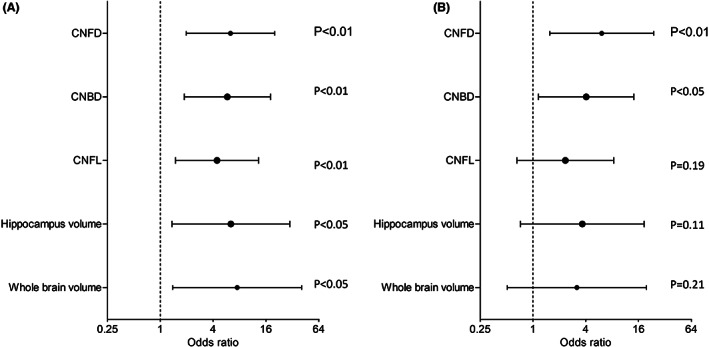
Predictors for progression to dementia in patients with mild cognitive impairment. The odds ratios, 95% CI of corneal nerve fiber density (CNFD), branch density (CNBD), fiber length (CNFL), intracranial volume of hippocampus, and whole brain adjusted for (A) hypertension, triglycerides, HDL, and duration of diabetes, and (B) age, cognitive function, hypertension, triglycerides, HDL, and duration of diabetes.

The OR for progression to dementia after adjusting for age, MoCA, hypertension, triglycerides, HDL and diabetes duration remained 6.1 (95% CI: 1.6–23.8) times higher with abnormal CNFD and 4.1 (95% CI: 1.2–14.2) times higher with abnormal CNBD but was no longer significant with abnormal CNFL (2.3, 95% CI: 0.7–8.3, *p* = 0.19), hippocampus (3.7, 95% CI: 0.6–22.5, *p* = 0.16), and whole brain volume (5.4, 95% CI: 0.7–44.6, *p* = 0.12) (Table 3 and Fig. 2). The sensitivity analysis where missing MRI data were coded as unknown (*n* = 28, 26.2%) gave similar results as the analysis where the missing data were ignored for hippocampus (3.7, 95% CI: 0.7–18.5, *p* = 0.12), and whole brain volume (3.2, 95% CI: 0.5–19.7, *p* = 0.22).

**Table 3:** The adjusted odds ratios for progression from MCI to dementia.

Variables	Cutoff scores	AOR	95% CI	*p* value
Abnormal CNFD (no./mm^2^)	≤25	6.1	1.6–23.8	0.01
Abnormal CNBD (no./mm^2^)	≤44	4.1	1.2–14.2	<0.05
Abnormal CNFL (mm/mm^2^)	≤15	2.3	0.7–8.3	0.19
Abnormal hippocampus (ICV%)	≤0.44	3.7	0.6–22.5	0.16
Abnormal hippocampus (ICV%)^a^	≤0.44	3.7	0.7–18.5	0.12
Abnormal whole brain (≤72 ICV%)	≤72	5.4	0.7–44.6	0.12
Abnormal whole brain (≤72 ICV%)^a^	≤72	3.2	0.5–19.7	0.22

Adjusted odds ratios (AOR), their corresponding 95% confidence intervals (CI) and *p* value are presented. Progression to dementia from MCI was adjusted for age, MoCA, hypertension, triglycerides, high‐density lipoprotein (HDL), and duration of diabetes.

^a^ Result of sensitivity analysis where the multiple logistic regression analysis was repeated with missing data for categorical MRI variables coded as unknown.

Table S1 shows that there were no differences in corneal confocal microscopy measures and volume of whole brain and hippocampi between participants with MCI with and without diabetes.

## Discussion

This study shows that people with MCI have evidence of corneal nerve loss, which was more severe in those who progressed to dementia. Furthermore, corneal nerve loss but not brain volumetry, was independently associated with progression to dementia. Corneal confocal microscopy (CCM), an ophthalmic marker of neurodegeneration has higher prognostic accuracy than MRI brain assessment for predicting progression from MCI to dementia. This provides further support for the utility of CCM in addition to its ability to identify neurodegeneration in people with MCI and dementia and better diagnostic capability compared to MRI brain volumetry.^15^, ^16^

In a Cochrane review,^29^ the overall predictive accuracy for the volume of hippocampus or whole brain for identifying progression from MCI to AD showed a wide range of sensitivities (28%–100%) and specificities (41%–100%). In the current study, hippocampal volume had a higher sensitivity (86%) and comparable specificity (62%) to predict the development of dementia in people with MCI compared to most studies,^28^, ^30^ A European study^28^ of 328 people with MCI aged 70.6 ± 7.6 years followed for 2‐years reported 78% sensitivity and 65% specificity for identifying the 28% who progressed to AD. A US, Canadian and European study^30^ of 447 participants with MCI aged 74.9 ± 6.9 years followed over 1‐year, reported a 77% sensitivity and 50% specificity for identifying the 20% who progressed to AD. We speculate that the lower mean age of our MCI cohort, longer mean follow‐up of 2.5‐years, and higher magnetic field strength of 3 versus 1.5 Tesla MRI compared to previous studies^28^, ^30^ may explain the higher predictive accuracy of hippocampal volume in identifying progression to dementia.

To date, there is no single biomarker with a high sensitivity and specificity for predicting progression from MCI to dementia. Indeed, CCM has a low sensitivity (55%–67%), but high specificity (78%–84%), thus an abnormal corneal nerve measure predicts progression from MCI to dementia with a low false positive rate. The low sensitivity may be partially attributed to the short follow‐up period of 2‐years or less in half of the study cohort, of which one third had abnormal CCM measures who may well have developed dementia with a longer duration of follow up. Nevertheless, the high negative predictive values (80%–84%) for CCM indicate that it could be used to identify patients with MCI who have a very low probability of progressing to dementia. Certainly, CCM has an independent prognostic ability compared to hippocampus and whole brain volume. Combining CCM measures with brain volumetry did not have any major impact.

Most research has focused on exploring new biomarkers for AD, however “pure” AD is not the most prevalent type of dementia in patients above the age of 70. We previously reported 44% mixed AD with vascular lesions, 32% AD, and 24% vascular dementia (VaD) in a cohort aged 73.4 years.^14^ Several risk factors for dementia,^5^, ^6^, ^7^, ^8^, ^9^, ^14^ including age, vascular disease, diabetes, hypertension, obesity, dyslipidemia, and sleep deprivation may limit the diagnostic accuracy of biomarkers for AD.^7^, ^10^, ^11^ Cortical and subcortical ischemia, risk factors for dementia, are associated with corneal nerve loss, brain atrophy, and cognitive deficits in MCI and dementia.^7^ Diabetes, a risk factor for dementia,^8^, ^9^ is independently associated with corneal nerve loss.^31^ This study shows that diabetes duration, hypertension, triglycerides, and HDL were significant covariates for progression from MCI to dementia. Interestingly, after adjusting for these covariates progression to dementia from MCI remained significantly associated with abnormal CCM measures, volume of hippocampus and whole brain. However, after further adjustment for age and cognitive function, progression to dementia remained significantly associated with abnormal CNFD and CNBD, but not with abnormal brain volume. This suggests that corneal nerve loss is a more direct measure of early neurodegeneration in MCI, whilst the loss of brain volume was associated with age but not with progression to dementia. Both corneal nerve fiber density and nerve fiber length are reliable measures of neurodegeneration in MCI and can distinguish those who progress to dementia from those who remain with MCI. The lower branch‐to‐fiber ratio in patients with MCI who progressed to dementia, suggests that impaired nerve regeneration may precede the development of dementia.

In this study, about half the patients with MCI reverted to normal cognition, consistent with previous studies reporting 46%–59% reversion to normal cognition.^32^, ^33^ The high reversion rate may be attributed to a younger age, no history of cerebrovascular disease, the APOE4 genotype, and better control of vascular risk factors.^32^, ^33^, ^34^ It could also be attributed to a false positive diagnosis of MCI in those who reverted, as the diagnosis of MCI in this study was based on ICD‐10 criteria, without confirmation using AD biomarkers. This study shows that whilst participants with MCI who reverted to NCI had lower corneal nerve measures compared to those with NCI they had marginally higher corneal nerve measures and comparable whole brain and hippocampal volumes compared to those who remained with MCI. This suggests that participants with MCI who remain with MCI or revert to normal cognition have less severe neurodegeneration compared to those with MCI who progress to dementia.

We acknowledge the relatively small cohort size, short duration of follow up and the lack of more rigorous measures and biomarkers of AD, including cortical Aβ‐42 or tau PET, CSF Aβ‐42 or phosphorylated tau, CSF T‐tau or FDG PET hypometabolism.^3^ The inclusion of these biomarkers of AD might give us a better understanding of the accuracy and reliability of CCM as a prognostic biomarker of dementia. Although a median follow‐up of 2.4 years may provide a reasonable estimate of the conversion rate from MCI to dementia, the wide follow‐up range of 1–4.5 years may limit accurate evaluation of the predictive capability of CCM. This study sets a foundation for larger studies to validate the utility of CCM in dementia, using rapid automated image nerve analysis software such as ACCMetrics or artificial intelligence (AI)‐based deep learning algorithm (DLA). As this is the first study to determine the prognostic ability of corneal nerve measures for the development of dementia in people with MCI, we have taken a more restrictive approach in relation to inclusion criteria to exclude corneal dystrophies or other neuropathies which are not typical comorbid conditions in people with MCI. However, we have included diabetes, a risk factor for corneal nerve degeneration and dementia, which is highly prevalent in patients attending our geriatric and memory clinic. We acknowledge that future research with a larger more heterogeneous cohort is required to study the generalizability of the results and obtain truly translational results. This is an ongoing study which will compare the trajectory of change in CCM to brain volumetry in individuals with NCI who do and do not progress to MCI, and individuals with MCI who do and do not progress to dementia. Another area worth exploring with CCM is alterations in dendritic cell density and morphology in the sub‐basal nerve layer as a potential biomarker immune mediated neurodegeneration in MCI and dementia. A preliminary report^35^ has shown that patients with MCI have different dendritic cell morphology compared to controls.

The current consensus is that early intervention may have a greater impact on neurodegeneration in people with MCI to prevent or delay the development of dementia.^29^ CCM may be useful to identify neurodegeneration in individuals presenting with cognitive impairment and could be used to select individuals with early or more progressive neurodegeneration, enabling enrichment in clinical trials of neuroprotective or disease‐modifying therapies.

## Author Contributions

Malik, Al Hamad and Ponirakis had full access to all the data in the study and take responsibility for the integrity of the data and the accuracy of the data analysis. Study concept and design: Malik and Ponirakis. Acquisition, analysis, or interpretation of data: All authors. Drafting of the manuscript: Ponirakis and Malik. Critical revision of the manuscript for important intellectual content: All authors. Statistical analysis: Ponirakis, Mahfoud and Malik. Obtained funding: Malik, Al Hamad. Administrative, technical, or material support: Malik, Al Hamad, Ponirakis, Gadelseed, Arasn, Tosino and Elorrabi.

## Conflict of Interest Statement

The authors declare that they have no competing interests.

## Funding Information

This publication was made possible by NPRP‐Standard (NPRP‐S) Twelfth (12th) Cycle grant # NPRP12S‐0213‐190080 from the Qatar National Research Fund (a member of Qatar Foundation). The findings herein reflect the work and are solely the responsibility of the authors.

## Supporting information


Supplementary Figure 1.

Supplementary Table 1.
