# Supplementary figures and images for: Corneal nerve loss predicts dementia in patients with mild cognitive impairment
Source: Ann Clin Transl Neurol. 2023 Feb 28;10(4):599–609. doi: 10.1002/acn3.51747 (PMC10109317; doi:10.1002/acn3.51747)

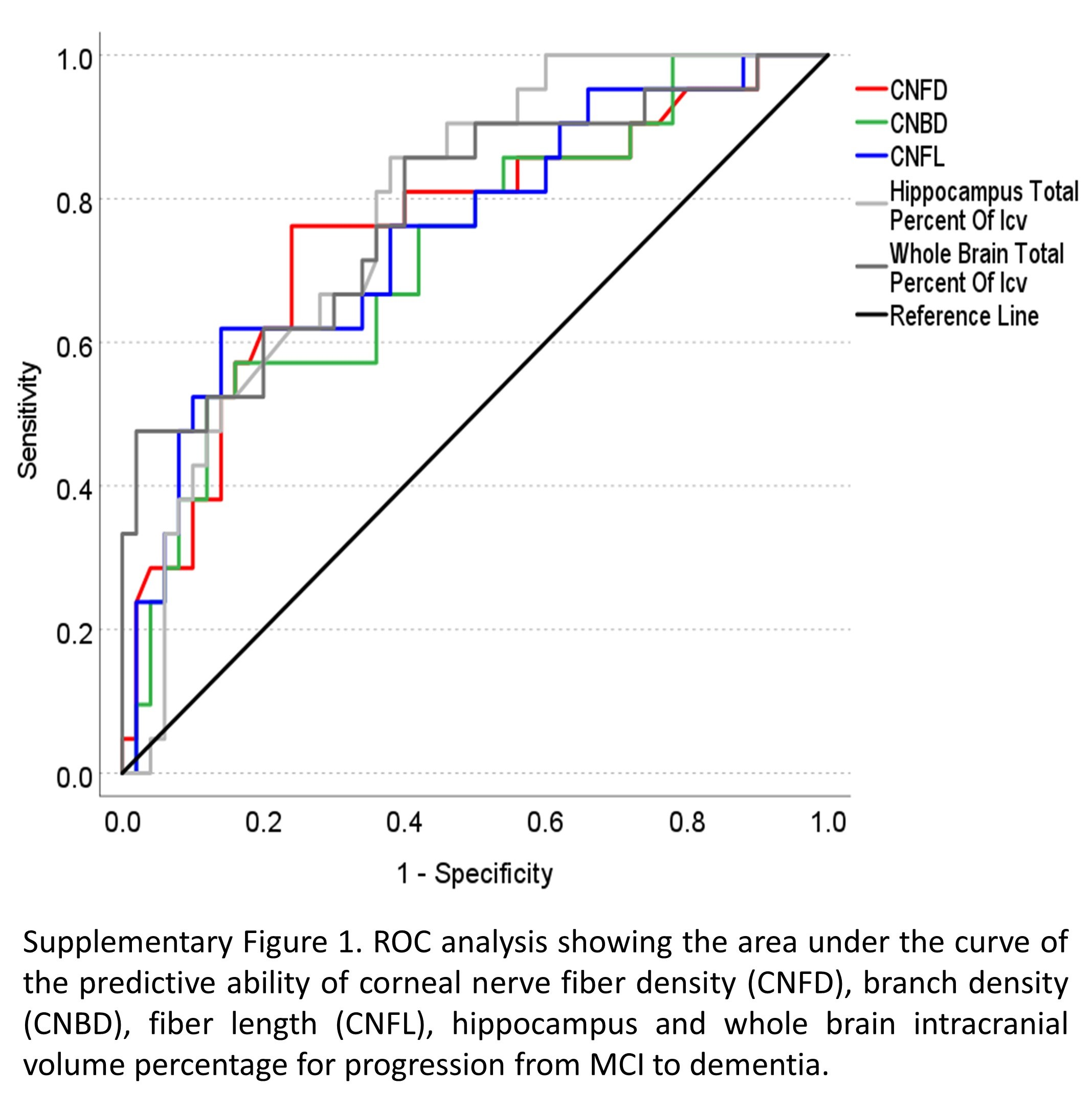

Supplement: Supplementary file 1 — Supplementary Figure 1. [file ACN3-10-599-s001.tif]
