# Supplementary material for: Corneal nerve loss predicts dementia in patients with mild cognitive impairment
Source: Ann Clin Transl Neurol. 2023 Feb 28;10(4):599–609. doi: 10.1002/acn3.51747 (PMC10109317; doi:10.1002/acn3.51747)
Supplement: Supplementary file 2 — Supplementary Table 1. [file ACN3-10-599-s002.docx]

**Supplementary table 1.** Comparison of corneal confocal microscopy measures and volume of whole brain and hippocampi between patients with MCI with and without diabetes.

|  | MCI without diabetes | MCI with diabetes | P value |
| --- | --- | --- | --- |
| n (%) | 52 (48.6) | 55 (51.4) |  |
| CNFD, fibers/mm^2^ | 27.6±8.8 | 24.9±9.6 | 0.13 |
| CNBD, branches/mm^2^ | 63.4±39.1 | 61.2±35.1 | 0.76 |
| CNFL, mm/mm^2^ | 18.7±6.3 | 17.7±6.4 | 0.42 |
| CNBD/CNFD ratio | 2.2±1.1 | 2.4±1.1 | 0.46 |
| Whole Brain, ICV% | 72.06±3.99 | 70.98±3.22 | 0.22 |
| Hippocampus, ICV% | 0.43±0.09 | 0.43±0.08 | 0.88 |

Variables presented as mean ± standard deviation were compared using unpaired t-test. Abbreviations: Mild cognitive impairment (MCI), corneal nerve fiber density (CNFD); corneal nerve branch density (CNBD), corneal nerve fiber length (CNFL), intracranial volume (ICV).
